# Uniform and Conformal Carbon Nanofilms Produced Based on Molecular Layer Deposition

**DOI:** 10.3390/ma6125602

**Published:** 2013-12-02

**Authors:** Peng Yang, Guizhen Wang, Zhe Gao, He Chen, Yong Wang, Yong Qin

**Affiliations:** 1 State Key Laboratory of Coal Conversion, Institute of Coal Chemistry, Chinese Academy of Science, Taiyuan 030001, China; E-Mails: yangpeng@sxicc.ac.cn (P.Y.); wangguizhen0@hotmail.com (G.W.); gaozhe@sxicc.ac.cn (Z.G.); 2 University of Chinese Academy of Sciences, Beijing 100039, China; 3 Key Laboratory of Chinese Education Ministry for Tropical Biological Resources, Hainan University, Haikou 570228, China; 4 State Key Laboratory of Materials-Oriented Chemical Engineering, College of Chemistry and Chemical Engineering, Nanjing University of Technology, Nanjing 210009, China; E-Mails: heizai.1990@163.com (H.C.); yongwang@njut.edu.cn (Y.W.)

**Keywords:** carbon nanofilm, molecular layer deposition, polyimide, pyrolysis

## Abstract

Continuous and uniform carbon nanofilms (CNFs) are prepared by pyrolysis of polyimide films which are produced by molecular layer deposition (MLD). The film thickness can be easily controlled at nanometer scale by altering the cycle numbers. During the annealing process at 600 °C, the polyimide film is subject to shrinkage of 70% in thickness. The obtained CNFs do not exhibit a well-graphitized structure due to the low calcination temperature. No clear pore structures are observed in the produced films. CNFs grown on a glass substrate with a thickness of about 1.4 nm shows almost 98% optical transmittance in the visible spectrum range. Au nanoparticles coated with CNFs are produced by this method. Carbon nanotubes with uniform wall thickness are obtained using anodic aluminum oxide as a template by depositing polyimide films into its pores. Our results demonstrate that this method is very effective to coat conformal and uniform CNFs on various substrates, such as nanoparticles and porous templates, to produce functional composite nanomaterials.

## 1. Introduction

Carbon films are attracting significant interest from researchers due to their excellent properties and they are widely used in catalysis, gas separation, analysis, energy storage and wear resistant coatings [1,2,3,4,5,6], *etc*. Carbon films would be superior to silica or alumina films due to their better chemical stability and biocompatibility, thus have special applications as protective or biocompatible layers [7]. Carbon-encapsulated nanomagnets were used as supports for catalytically active species and allowed for direct handling in acidic solutions due to the protection of carbon layers [5]. Zhang *et al*. [8] produced Cu_2_O nanowire arrays protected by carbon layers which show notably improved photostability and water splitting performance. Various methods have been exploited to grow carbon films, such as plasma enhanced chemical vapor deposition, pulsed laser deposition, sputtering technique, filtered cathodic jet carbon arc technique, pyrolysis of polymeric materials, and solution-based carbon precursor coating process [8,9,10,11,12,13,14,15,16,17,18,19,20,21], *etc*. However, these conventional methods have difficulty in meeting the requirement of precise control over the thickness of produced carbon films, require complicated procedures, and tend to cause fractures in the derived carbon films. Moreover, these methods are not suitable to produce uniform, continuous and conformal carbon films on substrates with a complex morphology such as porous nanomaterials.

Many works have been focusing on pyrolysis or graphitization of polyimide at high temperature to get carbon materials [22,23,24]. In this work, we apply molecular layer deposition (MLD) to get polyimide films [25]. Then carbon nanofilms (CNFs) are obtained by pyrolyzing the polyimide films under a protective H_2_/Ar atmosphere. As a variant of atomic layer deposition (ALD), MLD is developed for the growth of organic and hybrid organic-inorganic polymers with similar self-limiting surface reactions to that of ALD. The self-limiting surface reactions ensure MLD has advantages of precise thickness control at monolayer level, excellent step coverage and good conformity on complex substrates. Thus, it can be expected that the CNFs prepared through pyrolysis of polyimide films deposited by MLD will offer precisely tunable thickness and be of high quality.

## 2. Experimental Section

### 2.1. Synthesis of CNFs

The MLD of polyimide was carried out in a home-made, closed type, hot-wall ALD reactor. The deposition was carried out with ethylenediamine (EDA) and 1,2,4,5-benzenetetracarboxylic anhydride (PMDA) as precursors using N_2_ as a carrying gas [25]. The deposition temperature was 165 °C and EDA and PMDA were kept at room temperature and 150 °C, respectively. The obtained polyimide films were then transferred into a quartz tube furnace and annealed at 600 °C for 2 h under protecting H_2_/Ar gas flow at normal pressure to produce CNFs.

### 2.2. Preparation of Au Nanoparticles Coated with CNFs and Tubular Carbon Nanofibers

Carbon supported Cu grid was firstly coated with a 2 nm Al_2_O_3_ layer by ALD [26], then 1 µL solution of spherical gold nanoparticles with an average diameter of 50 nm (Strem Chemicals Inc., Newburyport, MA, USA) was dropped on this Cu grid protected with Al_2_O_3_ layer. After being air dried, it was deposited with polyimide films. Au nanoparticles coated with CNFs (referred to as Au/C hereafter) were finally produced by annealing according to the procedure mentioned above.

Anodic aluminum oxide (AAO) template with a 200 nm pore diameter was also used as a substrate for the deposition of polyimide films. After the deposition of polyimide, the sample was annealed at 600 °C for 2 h under protecting H_2_/Ar gas. Then the sample was immersed into 1 M NaOH aqueous solution at 45 °C for 2 h to etch away the AAO template. The black residuals were rinsed with deionized water several times. The samples eventually obtained were carbonaceous nanotubes (referred to as C-NTs hereafter).

### 2.3. Characterization of CNFs

Raman spectra of the samples were collected on a Horiba Labram HR800 (Horiba Jobin Yvon, France) spectrometer with an excitation wavelength of 633 nm. The chemical composition and bonding configurations of the prepared samples were evaluated by X-ray photoelectron spectroscopy (XPS) (Kratos Analytical, Ltd., Manchester, UK) performed on a Kratos XSAM 800 spectrometer using Al Kα (hk = 1486.6 eV) X-ray source. The shrinkage ratio of the films after annealing was analyzed from cross-sectional samples by scanning electron microscopy (SEM) on a Hitachi S-4800 microscope (Hitachi, Ltd., Tokyo, Japan). The surface roughness of the samples was observed by atomic force microscopy (AFM) on a CSPM 5500 scanning probe microscope (Being Nano-Instruments, Ltd., Guangzhou, China). The optical transmittance of the CNFs produced on quartz glass substrates was measured by ultraviolet-visible spectroscopy (UV-Vis) on a Hitachi U-3900 spectrophotometer (Hitachi High-Technologies Corporation, Tokyo, Japan). Transmission electron microscopy (TEM) was applied to observe the microstructure of Au/C nanoparticles and C-NTs using a JEOL-2100F microscope (JEOL Ltd., Tokyo, Japan). The TEM specimen of C-NTs was prepared by drying a droplet of C-NTs-containing ethanol suspension on a TEM Cu grid.

## 3. Results and Discussion

### 3.1. Structure Analysis and Properties of CNFs

**Figure 1:**
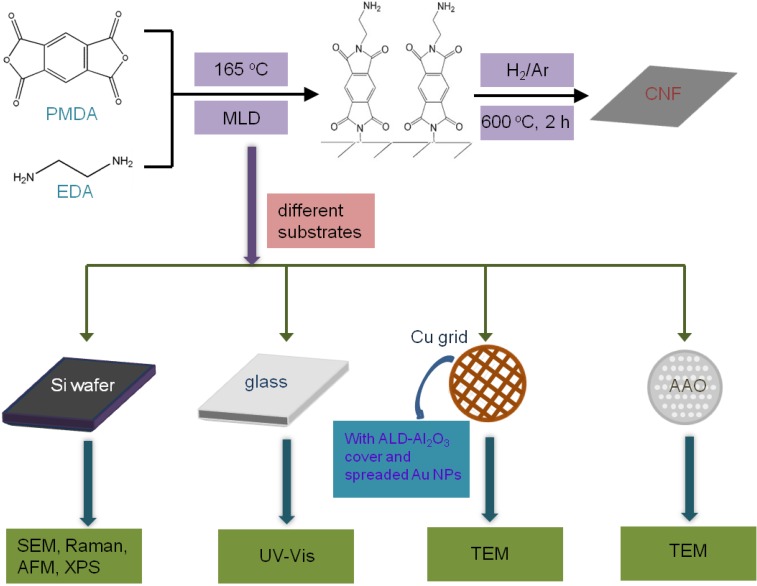
Scheme of the experimental process.

**Figure 2:**
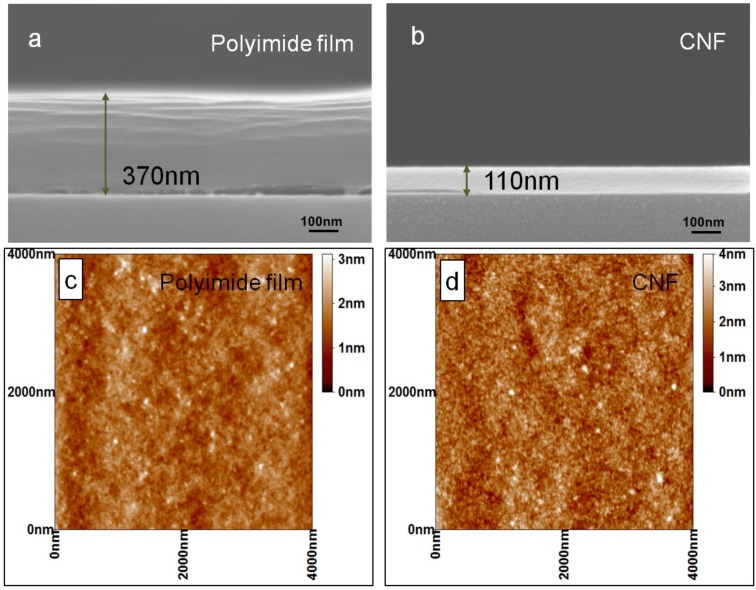
Cross-sectional Scanning electron microscope (SEM) images (**a**,**b**) and Atomic force microscope (AFM) analysis (**c**,**d**) of polyimide film and carbon nanofilm (CNF).

A schematic shown in Figure 1 displays the experimental process of the preparation and analysis of the CNFs. The CNFs are produced on different substrates for different characterizations. For SEM investigation, polyimide films were firstly deposited on a Si wafer with 800 MLD cycles. The large cycle number was chosen for precise and convenient thickness measurement by SEM. Two cross-sectional samples were created by cleavage of a polyimide/Si wafer. Then one sample was converted into CNF/Si through heat treatment under the conditions mentioned above. The SEM images of cross-sectional samples of polyimide film and CNF are shown in Figure 2a,b, respectively. Note that the polyimide film is slightly separated from the Si substrate, which can be ascribed to the dragging force when the Si substrate was cut to make the cross-sectional samples. The cross-section of CNF is flatter and more regular than that of polyimide film due to higher strength. The thicknesses of the polyimide film and CNF are about 370 and 110 nm, respectively. Accordingly, the growth rate of polyimide film is calculated to be 4.6 Å per MLD cycle, and correspondingly each MLD cycle can increase the thickness of CNF by 1.4 Å after annealing. The shrinkage ratio is about 70% during the heat treatment. AFM measurement was employed for further investigation of the surface roughness of the samples. Figure 2c,d displays the AFM images of polyimide film and CNF within an area of 3 × 3 μm^2^. The surface roughness analysis reveals that the root-mean-squared roughnesses are 0.30 and 0.44 nm for polyimide film and CNF, respectively. The AFM investigation reveals that the surface of CNF is very smooth, though it is slightly rougher than the as-deposited polyimide film.

**Figure 3:**
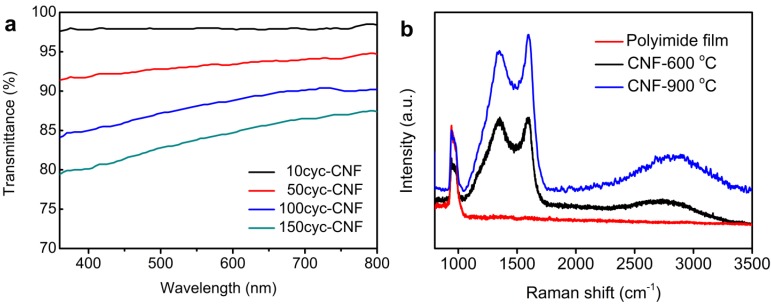
UV-Vis (**a**) and Raman (**b**) spectra of CNF samples.

The optical transmittance of the CNFs coated on glass substrates are studied by UV-Vis measurement with a bare glass wafer as reference. Figure 3a shows the UV-Vis spectra of CNFs with different thicknesses. The CNF synthesized with 10 MLD cycles (about 1.4 nm according to the shrinkage ratio mentioned above) exhibits almost 98% transmittance in the visible spectral range, indicating that the CNF is almost transparent. The structural feature of the CNFs was further analyzed by Raman spectroscopy. Raman analysis is a widely used, non-destructive way to characterize the bonding structure of various carbonaceous materials [27]. Two bands are observed from the Raman spectrum of the CNF prepared by annealing polyimide films at 600 °C (Figure 3b) at 1354 and 1594 cm^−1^, which can be assigned to D and G bands of carbon materials, respectively, while neither of these two peaks appears in the Raman spectrum of polyimide films. The D band is considered to represent the disorder-induced features caused by lattice defect [28,29], and the G band represents the E_2g_ vibrational mode within aromatic carbon rings [28,29,30]. The occurrence of the G band in the Raman spectrum of the CNF implies that the heat treatment produces some graphitized domains in the sample. The intensity ratio value of I(D)/I(G) is usually used to depict the degree of graphitization of carbon materials [31]. The calculated I(D)/I(G) of CNF is 0.99, revealing that the CNFs are not completely graphitized under the annealing conditions mentioned above. The graphitization of the CNF can be improved by increasing the calcination temperature. The CNF prepared by annealing polyimide films at 900 °C was also investigated with Raman spectroscopy. Its I(D)/I(G) value is about 0.93, indicating that the higher annealing temperature results in better graphitized structure. The D band in the Raman spectrum of CNFs may be caused by the structural defects or the residue of some impurity atoms after the pyrolysis process, which is also revealed by XPS results below.

**Figure 4:**
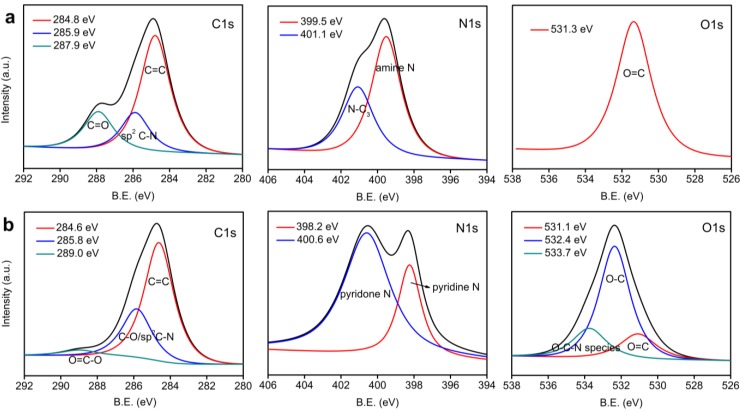
XPS spectra of polyimide film (**a**) and CNF (**b**).

The contents of C, N and O calculated from XPS measurements for polyimide films and CNFs are summarized in Table 1. After heat treatment under H_2_/Ar atmosphere at 600 °C, the content of C increases while the contents of O and N decrease, indicating the decomposition and removal of groups containing O or N. Figure 4a,b show the high-magnification XPS spectra of polyimide film and CNF, respectively. Compared with polyimide film, the C1s spectrum of CNF shows an enhanced peak at 285.8–285.9 eV which can be ascribed to C–O or sp^2^ C–N bonds [32]. C=O bond does not appear in the C1s spectrum of CNF. These results indicate that the C=O groups are reduced to C–O, which is also confirmed by the change of O1s during the annealing process according to the O1s spectra of polyimide film and CNF. From the N1s spectra of polyimide film and CNF, it can be observed that amine N (at around 399.5 eV) and quaternary N (at around 401.1 eV) [33] does not exist after heat treatment, while pyridine N and pyridone N appear at around 398.2 and 400.6 eV [33]. This confirms the formation of pyridinic N during pyrolysis [34]. This result implies that the CNFs may find applications in catalysis because pyridinic nitrogen is an important part of the electrocatalytic active sites [35].

**Table 1:** Elemental composition of polyimide film and CNF.

Sample	C	N	O
Polyimide film	66.66%	12.44%	20.90%
CNF	81.49%	4.72%	13.79%

### 3.2. Au/C Nanoparticles and C-NTs

**Figure 5:**
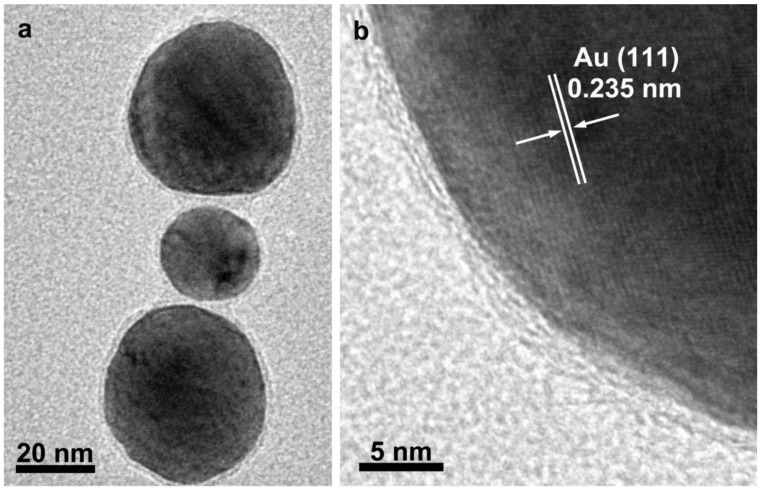
TEM (**a**) and HRTEM (**b**) images of Au/C nanoparticles.

Au nanoparticles coated with CNFs were successfully produced by our method. The morphology of the obtained samples was characterized with TEM. Figure 5a shows the core-shell structure of the hybrid Au/C nanoparticles. It can be clearly seen that each Au nanoparticle is coated with a uniform carbon shell. The thicknesses of the carbon shells around each particle are identical. Figure 5b is a high resolution TEM (HRTEM) image for an individual Au/C nanoparticle. In the core region, the lattice fringes with spacings of 2.35 Å are clearly visible corresponding to Au (111) plane. Surrounding the Au core, a thin carbon layer of about 2 nm thickness is observed. The protecting carbon layers prevented the aggregation of neighboring Au nanoparticles during the annealing process.

**Figure 6:**
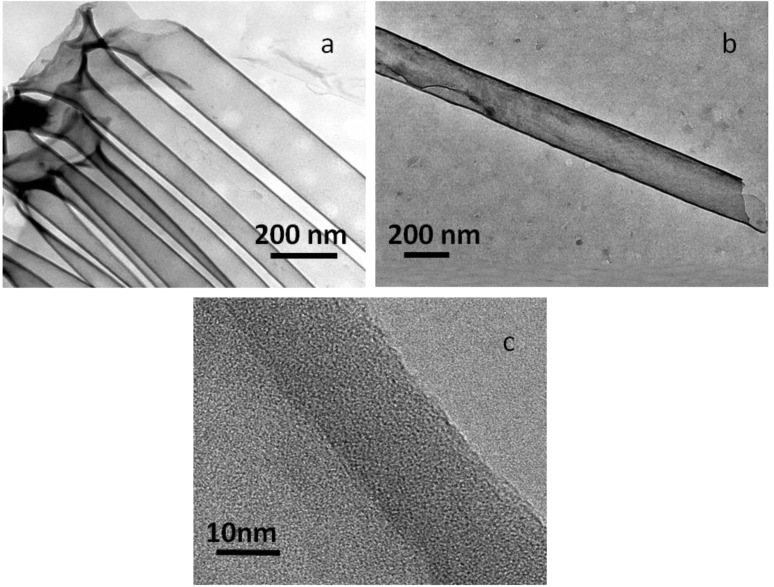
TEM (**a**,**b**) and HRTEM (**c**) images of C-NTs.

Figure 6a is a typical TEM image of C-NTs prepared using AAO as a template as mentioned in the experimental section. The outer diameter of the C-NTs is about 200 nm which is consistent with the pore diameter of AAO template. The C-NTs are over 10 µm in length and their tube walls are very smooth with a uniform thickness of about 15 nm (Figure 6b). No pore in the walls can be observed from the HRTEM image of one individual tube as shown in Figure 6c. The C-NTs maintain a tubular structure well after treatment with NaOH solution during the etching process for the removal of AAO template, indicating the CNFs have very good chemical stability. No lattice fringes of graphite can be clearly seen from the HRTEM image. This further reveals that the tube walls are not well graphitized due to the low annealing temperature, consistent with the Raman analysis.

## 4. Conclusions

In this work, a new strategy for the preparation of continuous and uniform CNFs has been demonstrated. This is achieved by pyrolyzing polyimide films deposited by MLD. The thickness of the CNFs can be precisely and conveniently controlled by adjusting the cycle numbers. The CNFs have very good optical transparency. Au/C core-shell nanoparticles and C-NTs are produced by this method. Our method is suitable for the synthesis of functional nanomaterials by coating CNFs with uniform, conformal and precisely controlled thickness on various nanostructures even with complex morphology.
